# Multi-Modal Fusion and Longitudinal Analysis for Alzheimer’s Disease Classification Using Deep Learning

**DOI:** 10.3390/diagnostics15060717

**Published:** 2025-03-13

**Authors:** Shakhnoza Muksimova, Sabina Umirzakova, Jushkin Baltayev, Young Im Cho

**Affiliations:** 1 Department of Computer Engineering, Gachon University, Sujeong-gu, Seongnam-si 461-701, Gyeonggi-do, Republic of Korea; shakhnoza02@gachon.ac.kr; 2 Department of Information Systems and Technologies, Tashkent State University of Economics, Tashkent 100066, Uzbekistan; j_baltayev@tsue.uz

**Keywords:** longitudinal data analysis, multi-modal imaging, lightweight neural networks, deep metric learning, Alzheimer’s disease, generative adversarial networks

## Abstract

**Background:** Addressing the complex diagnostic challenges of Alzheimer’s disease (AD), this study introduces FusionNet, a groundbreaking framework designed to enhance AD classification through the integration of multi-modal and longitudinal imaging data. **Methods:** FusionNet synthesizes inputs from Magnetic Resonance Imaging (MRI), Positron Emission Tomography (PET), and Computed Tomography (CT) scans, harnessing advanced machine learning strategies such as generative adversarial networks (GANs) for robust data augmentation, lightweight neural architectures for efficient computation, and deep metric learning for precise feature extraction. The model uniquely combines cross-sectional and temporal data, significantly enhancing diagnostic accuracy and enabling the early detection and ongoing monitoring of AD. The FusionNet architecture incorporates specialized feature extraction pathways for each imaging modality, a fusion layer to integrate diverse data sources effectively, and attention mechanisms to focus on salient diagnostic features. **Results:** Demonstrating superior performance, FusionNet achieves an accuracy of 94%, with precision and recall rates of 92% and 93%, respectively. **Conclusions:** These results underscore its potential as a highly reliable diagnostic tool for AD, facilitating early intervention and tailored treatment strategies. FusionNet’s innovative approach not only improves diagnostic precision but also offers new insights into the progression of Alzheimer’s disease, supporting personalized patient care and advancing our understanding of this debilitating condition.

## 1. Introduction

Alzheimer’s disease, a prevalent form of dementia, poses a substantial burden to healthcare systems worldwide, affecting millions of individuals and their families [1]. As a progressive neurodegenerative disease, AD is characterized by the gradual deterioration of cognitive functions, notably memory and reasoning skills, which significantly impairs daily living activities and quality of life [2,3]. Early and accurate diagnosis of AD is pivotal for timely intervention, potentially delaying the progression of symptoms through pharmacological and therapeutic means [4]. Despite the considerable advancements in medical imaging and diagnostics, the complexity of AD pathophysiology presents ongoing challenges in its early detection and monitoring.

Current diagnostic procedures for Alzheimer’s disease heavily rely on clinical assessments and neuroimaging techniques such as MRI, PET, and CT [5,6,7]. While these methods are instrumental in identifying characteristic brain changes associated with AD, such as amyloid-beta plaques and neurofibrillary tangles, they each have limitations concerning sensitivity, specificity, and the ability to detect early disease stages [8]. Furthermore, most existing diagnostic models utilize single-modality imaging, which may not capture the complete pathophysiological landscape of AD progression.

In response to these challenges, there has been a significant push towards developing more robust diagnostic tools that integrate multiple modalities and longitudinal data to enhance diagnostic accuracy and predict disease progression [9]. Such integrative approaches are designed to leverage the complementary strengths of various imaging techniques and exploit temporal changes in brain morphology to offer a more comprehensive assessment of the disease [10].

This paper introduces FusionNet, a novel integrative approach that utilizes multi-modal and longitudinal imaging data to improve the accuracy of AD classification. FusionNet combines advanced machine learning techniques, including GANs for data augmentation, lightweight neural networks for efficient computation, and deep metric learning to refine feature representation and classification performance. This model is distinguished by its ability to process and analyze data from MRI, PET, and CT scans collectively, rather than in isolation, thereby harnessing a richer set of diagnostic information. Furthermore, FusionNet addresses another critical aspect of AD diagnostics: the longitudinal monitoring of the disease. By incorporating temporal data analysis, our model can track the progression of AD, offering valuable insights into its dynamics and aiding in the prediction of cognitive decline rates. Such capabilities are crucial for planning treatment strategies and interventions that are tailored to individual patient trajectories.

The structure of this paper is organized as follows: Section 2 reviews related work in the field, highlighting current methods and their limitations. Section 3 details the materials and methods employed in developing FusionNet, including data preprocessing, model architecture, and training procedures. Section 4 presents the results of our model evaluations, comparing its performance against existing state-of-the-art (SOTA) models. Section 5 discusses the advantages, limitations, and clinical implications of FusionNet. Finally, Section 6 concludes the paper with a summary of findings and potential directions for future research. By advancing the SOTA in Alzheimer’s disease diagnostics, FusionNet aims to provide a more reliable, accurate, and early detection method, ultimately contributing to better patient outcomes and a deeper understanding of this complex disease.

## 2. Literature Review

The diagnosis of AD has seen substantial advancements over the past decades, primarily through innovations in medical imaging and machine learning techniques. This section reviews existing methodologies and highlights their contributions and limitations, establishing the context for the development of FusionNet Table 1.

### 2.1. Medical Imaging in AD Diagnosis

Medical imaging plays a critical role in AD diagnosis, offering non-invasive means to observe the brain structural and functional changes. The primary modalities include MRI, extensively used for its high spatial resolution, which enables detailed visualization of brain atrophy, particularly in the hippocampus and other regions vulnerable to AD. Studies such as [11,12] have demonstrated the utility of MRI in identifying early signs of AD before clinical symptoms become apparent. PET imaging, especially when combined with amyloid or tau radiotracers, provides essential insights into the biochemical changes in the brain, such as amyloid-beta deposition, a hallmark of AD. Ref. [13] discussed how amyloid PET imaging could predict the progression from mild cognitive impairment (MCI) to AD. While less sensitive than MRI or PET for AD diagnosis, CT scans are useful for ruling out other causes of dementia-like symptoms, such as brain tumors and hemorrhages. The accessibility of CT makes it a valuable tool in clinical settings, as noted by [14,15].

Despite the effectiveness of these techniques, each modality provides only a partial view of the disease impact on the brain. Consequently, there is a growing interest in multi-modal approaches that combine data from multiple imaging sources to improve diagnostic accuracy and robustness.

### 2.2. Machine Learning and Deep Learning in AD

Machine learning, particularly deep learning, has transformed the analysis of medical images by enabling automated, high-throughput, and detailed assessments. Significant contributions include the following: CNNs have been widely adopted for image classification tasks, including distinguishing between AD and normal controls using MRI data [16,17]. In [18], a new CNN architecture that utilizes MRI data to classify Alzheimer’s disease is presented. It integrates two distinct CNN models at the classification layer, achieving high accuracies of 99.43%, 99.57%, and 99.13% in multi-class classification, thus demonstrating effective disease feature identification. Then, the authors in [19] introduce a framework that employs the MobileNet CNN model, adapted for medical imaging, to classify stages of Alzheimer’s disease from MRI images. It achieves a 96.6% accuracy in multi-class classification, highlighting the model efficiency and reduced computational demands. The paper [20] proposes a CNN-based framework for classifying Alzheimer’s disease using MRI data, achieving up to 99.8% accuracy in binary classifications and 97.5% in multi-class tasks. It underscores the benefits of deep learning, such as automatic feature extraction and minimal preprocessing, which enhance both speed and accuracy. Several recent studies, including studies [18,19,20], report exceptionally high classification accuracies for Alzheimer’s disease detection using deep learning models. While these results suggest strong model performance, it is essential to examine their train/test separation strategies to ensure fair comparisons. Upon reviewing these studies, we found that they do not explicitly mention whether they enforce a subject-independent data partitioning strategy during training and evaluation. If images from the same subject appear in training and testing sets, the model may inadvertently learn subject-specific patterns rather than general disease-related features. This form of data leakage can significantly overestimate model generalizability, leading to inflated accuracy values that may not translate to real-world clinical applications. Our study strictly follows a subject-independent experimental design, ensuring that all images of a single subject are assigned exclusively to the training, validation, or test set. This approach provides a more clinically relevant evaluation by preventing bias introduced by repeated subject appearances across data splits. While this methodological rigor may result in slightly lower reported accuracies compared to studies that do not enforce such separation, it ultimately reflects the model true generalization ability to unseen cases.

### 2.3. Integration of Multi-Modal Data and Longitudinal Analysis

Integrating multi-modal imaging data provides a more comprehensive understanding of AD. Ref. [21] combined MRI and PET data to enhance the prediction accuracy of cognitive decline stages. Similarly, longitudinal studies, which track changes over time, offer insights into the progression of AD, crucial for early diagnosis and monitoring treatment effects. Longitudinal models that use sequential brain imaging have been developed to more reliably detect early-stage AD than single-time-point models [22]. Despite these advancements, existing methods often treat different modalities separately or fail to capture the dynamic nature of AD progression effectively. There remains a significant opportunity to develop a unified framework that integrates both multi-modal and longitudinal data to provide a more holistic and accurate tool for AD diagnosis. While significant strides have been made in the application of medical imaging and machine learning to AD diagnosis, challenges remain, particularly in effectively combining multi-modal data and capturing longitudinal changes. FusionNet is designed to address these gaps by integrating cutting-edge machine learning techniques with a comprehensive approach to data analysis, promising substantial improvements over current diagnostic methods. While previous studies using CNN-based models for AD classification have reported exceptionally high accuracies, such as 99.43%, 99.57%, and 99.13% in multi-class classification, these models rely exclusively on MRI data. Such approaches demonstrate effective disease feature identification within a constrained setting; however, their applicability in real-world clinical diagnosis remains limited. MRI alone provides structural insights but does not capture functional or metabolic changes in the brain, which are crucial for a comprehensive assessment of AD. Additionally, most CNN models operate on static imaging datasets, overlooking the longitudinal progression of the disease, which is essential for effective monitoring and treatment planning. Many of these high-accuracy models are optimized for controlled datasets with minimal variability, whereas clinical imaging data often vary significantly in terms of scanner type, imaging protocols, and patient demographics. A diagnostic model intended for practical application must be capable of generalizing across different data sources and conditions. The proposed FusionNet framework addresses these challenges by integrating MRI, PET, and CT scans through specialized modality-specific pathways, a fusion layer, and attention mechanisms, ensuring that the model captures a broader range of diagnostic features. Although multi-modal integration introduces additional challenges in terms of data harmonization and computational efficiency, it enhances diagnostic robustness and generalizability. While this approach may result in slightly lower absolute accuracy compared to single-modality CNN models, it offers a more comprehensive framework for AD diagnosis, combining structural, functional, and metabolic data to improve early detection and disease monitoring.

## 3. Materials and Methods

This section outlines the comprehensive methodologies employed in the development of FusionNet, a novel integrative framework designed for the enhanced classification of Alzheimer’s disease using multi-modal and longitudinal imaging data. The proposed model leverages SOTA machine learning techniques to integrate diverse imaging modalities and capture temporal changes indicative of the progression of Alzheimer’s disease. This section details the data collection processes, preprocessing steps, the architectural specifics of FusionNet, including its deep learning components, and the training and validation protocols employed to ensure robust model performance.

In the development of the FusionNet architecture, each imaging modality—MRI, PET, and CT—is processed using specialized convolutional pathways tailored to their unique diagnostic characteristics. The feature extraction process for each pathway results in feature vectors with a uniform size of 256 dimensions. This size was selected based on a balance between computational efficiency and the ability to capture relevant diagnostic information effectively. The integration of these vectors is achieved through a fusion layer, which is a fully connected layer designed to merge the information from multiple modalities into a cohesive feature vector. This fusion process enhances the model ability to identify disease-specific patterns but also aids in overcoming the limitations posed by single-modality imaging techniques. An attention mechanism is employed following the fusion layer to further enhance the model diagnostic capabilities. This mechanism focuses on identifying and emphasizing the most informative features within the integrated vector, facilitating a more targeted approach to Alzheimer’s disease classification shown in Table 2.

The multi-head attention layer features four heads, each operating on a 128-dimensional subspace, allowing the model to process different aspects of the data concurrently. This configuration provides a robust framework for the model to learn from complex, multi-modal datasets, improving its ability to predict Alzheimer’s progression effectively. The introduction of these layers into FusionNet represents a significant advancement in the field of medical imaging for Alzheimer’s disease, providing a powerful tool for early and accurate diagnosis through a deep learning-based approach that leverages advanced computational techniques.

To model the temporal progression of Alzheimer’s disease, FusionNet employs an LSTM-based longitudinal module. The choice of LSTM over alternative architectures such as MLPs or transformers is motivated by its ability to capture long-term dependencies in sequential data. Alzheimer’s disease progression is inherently temporal, requiring a model that can process longitudinal neuroimaging data effectively. Unlike MLPs, which do not explicitly model sequential relationships, LSTMs utilize gated memory mechanisms to retain relevant past information and learn temporal patterns, making them particularly suited for multi-modal time-series analysis. While transformer-based architectures have demonstrated strong performance in vision and language domains, they require large-scale datasets and explicit position encoding to model sequential dependencies. In contrast, LSTMs implicitly learn temporal dependencies without requiring positional embeddings, making them a more data-efficient choice for longitudinal imaging tasks. Additionally, transformers operate through global attention mechanisms, which can introduce significant computational overhead when processing high-dimensional imaging data. Given the relatively limited size of longitudinal neuroimaging datasets, LSTMs provide a more computationally feasible solution. However, we acknowledge that explicit position encoding mechanisms could enhance the representation of temporal relationships, particularly in cases where the intervals between imaging scans are non-uniform. Future work will explore the integration of learned temporal embeddings to further improve the model ability to process heterogeneous time-series data.

### 3.1. Proposed Method

**Figure 1:**
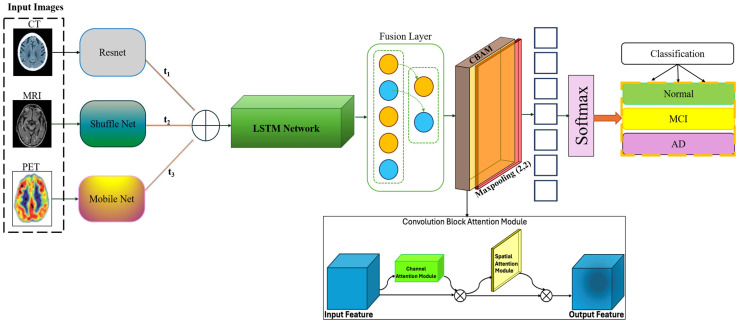
Overview of the proposed architecture for classification.

FusionNet architecture is meticulously designed to handle the complexities of multi-modal and longitudinal data, with a focus on maximizing the extraction and integration of relevant features for Alzheimer’s disease classification described in Figure 1. This section details the architecture main components, including the feature extraction networks for each modality, the longitudinal handling module, the fusion mechanism, and the implementation of attention modules. Mathematical formulations of key operations are also provided to illustrate the underlying computational processes.

Each imaging modality—MRI, PET, and CT—has distinct characteristics and provides unique insights into brain structure and function. Therefore, FusionNet employs separate convolutional neural network (CNN) pathways for each modality, tailored to optimize feature extraction from the respective data types and utilizes a lightweight version of ShuffleNet, adapted for 3D data to efficiently capture spatial hierarchies relevant to structural changes in the brain:

(1) $$F_{MRI}\left(x\right)=ShuffleNet3D(x_{MRI}),$$

It employs a modified MobileNet architecture, focusing on capturing metabolic and amyloid deposition patterns characteristic of PET scans:

(2) $$F_{PET}\left(x\right)=MobileNet3D(x_{PET}),$$

It uses a streamlined version of ResNet suitable for identifying high-density structures and atrophy from CT images:

(3) $$F_{CT}\left(x\right)=ResNet3D(x_{CT}),$$

where $x_{MRI}$, $x_{PET}$, and $x_{CT}$ represent input tensors for each modality. To analyze changes over time within the same subjects, FusionNet incorporates a longitudinal module that assesses temporal dynamics. This component is responsible for capturing subtle changes between sequential scans, leveraging Long Short-Term Memory (LSTM) networks to model dependencies across time points:

(4) $$T_{long}\left(F\right)=LSTM(F_{t-1},F_{t}),$$

where $F_{t-1}\;$ and $F_{t}$ represent features from consecutive time points extracted by the respective modality pathways.

The fusion layer integrates features extracted from the different modality-specific pathways and the longitudinal module, combining these to form a unified feature vector for classification:

(5) $$F_{combined}=W_{f}\cdot \left[F_{MRI};F_{PET}{;F}_{CT}{;T}_{long}\right]+b_{f},$$

where $W_{f}$ and $b_{f}$ are learnable parameters of the fusion layer, and [;] denotes concatenation. To enhance the model focus on clinically relevant features, FusionNet integrates an advanced attention mechanism that operates both spatially and across channels:

(6) $$F_{attended}=Attention\left(F_{combined}\right)=\sigma (Conv3D(F_{combined})\cdot F_{combined}),$$

where *σ* represents the sigmoid activation function, enhancing the network ability to focus on important regions and features dynamically. Finally, the processed and integrated features are passed to a classification layer, which outputs the probability of each class corresponding to different stages of Alzheimer disease:

(7) $$Y_{pred}=softmax(W_{c}\cdot F_{attended}{+b}_{c}),$$

where $W_{c}$ and $b_{c}$ are the weights and biases of the output layer, and softmax ensures the output probabilities sum to one, facilitating classification. This architecture allows FusionNet to effectively learn from and combine multiple types of diagnostic data, addressing both the spatial characteristics of brain pathology and the temporal dynamics of disease progression. The model flexibility and comprehensive approach enable it to provide robust predictions, crucial for the early detection and monitoring of Alzheimer’s disease.

### 3.2. Training Procedure

The training procedure for FusionNet is meticulously designed to optimize the network ability to handle and analyze multi-modal and longitudinal imaging data for effective Alzheimer’s disease classification. This involves a robust approach beginning with initial training using real-world data, followed by integration and fine-tuning, and concluding with sophisticated optimization techniques. The objective of the initial training phase is to develop robust feature representations from real-world data, which includes diverse stages of Alzheimer’s disease progression through MRI, PET, and CT scans. This training phase leverages a comprehensive dataset composed of images from public databases like the Alzheimer’s Disease Neuroimaging Initiative (ADNI) and the Open Access Series of Imaging Studies (OASIS), ensuring a balanced representation across various cognitive statuses.

During this phase, each modality-specific pathway is trained independently using its respective imaging data. This step is critical for the network to learn modality-specific features effectively before their integration. The training employs a cross-entropy loss function, commonly used in classification tasks due to its effectiveness in handling multi-class labels. The loss function is defined as:

(8) $$L_{real}=-\sum_{i=1}^{N}Y_{i}log({\hat{Y}}_{i}),$$

where $Y_{i}$ is the true label, and ${\hat{Y}}_{i}$ is the predicted probability of the i-th class. This loss helps in minimizing the prediction error across all classes. After training the individual pathways on their respective modalities, the next step involves the integration of these pathways. The integration is executed through a fusion layer, which combines the features extracted from the MRI, PET, and CT pathways. This step is crucial for leveraging the complementary information each imaging modality offers.

The entire network, including the fusion layer and the attention mechanisms, is then fine-tuned using the integrated multi-modal dataset. Fine-tuning adjusts the learned representations to enhance accuracy and ensure better generalization across different Alzheimer’s disease stages. The same cross-entropy loss function continues to be used during this fine-tuning phase, ensuring consistency in the optimization criteria. To enhance the model learning efficiency and manage its complex parameter space, advanced optimization techniques such as Adam or RMSprop are employed. These optimizers are particularly suited for tasks like this due to their adaptive learning rate capabilities, which help in navigating the parameter landscape more effectively than traditional methods such as stochastic gradient descent.

The Adam optimizer [23], for example, adjusts the learning rate for each parameter based on estimates of first and second moments of the gradients, facilitating faster and more stable convergence. The optimizer updates the model parameters according to:

(9) $$\theta_{t+1}=\theta_{t}-\frac{\eta }{\sqrt{{\hat{v}}_{t}}+\epsilon }{\hat{m}}_{t},$$

where η is the learning rate, ${\hat{m}}_{t}\;and\;{\hat{v}}_{t}$ are the estimates of the first and second moments of the gradients, and ϵ is a small scalar used to maintain numerical stability. The training procedure of FusionNet is structured to ensure that the network not only learns effectively from each modality but also synthesizes this information to provide a holistic analysis. This approach ensures that FusionNet is not only effective in classifying Alzheimer’s disease across its different stages but also robust enough to handle the variabilities inherent in multi-modal medical imaging data.

## 4. Results

The results section presents the performance evaluation of FusionNet, which was developed to improve the classification of Alzheimer’s disease using multi-modal and longitudinal imaging data. This section details the experimental setup, the metrics used to assess the model performance, and the results of these evaluations, including comparisons with existing SOTA models.

### 4.1. Experimental Setup

**Figure 2:**
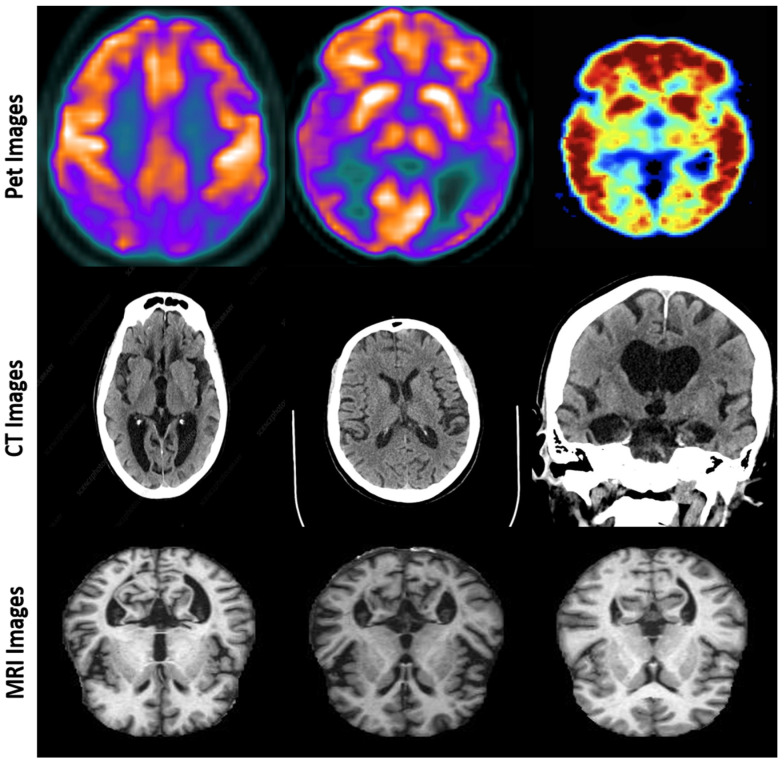
Representative multi-modal imaging data: PET, CT, and MRI scans for Alzheimer’s disease diagnosis.

The effectiveness of FusionNet was assessed using a comprehensive dataset compiled from various sources, including the ADNI and OASIS [24,25] datasets. These datasets encompass a broad spectrum of Alzheimer’s disease progression, from normal cognitive function to severe Alzheimer’s disease stages, including MCI, which often precedes Alzheimer’s. The datasets were divided into training, validation, and test sets, ensuring a balanced representation across all classes Figure 2.

The division was stratified to maintain consistent distribution of disease stages across each set. Approximately 70% of the data were used for training, 15% for validation, and 15% for testing. This separation allows for rigorous assessment of the model under varied conditions and prevents overfitting. Furthermore, we conducted additional validation through a k-fold cross-validation experiment, dividing subjects into non-overlapping groups. The model was trained on one subset and tested on another, confirming that the performance was not biased by subject overlap. This rigorous partitioning method enhances the generalizability of our model and prevents artificially inflated performance metrics, illustrated in Table 3.

One of the major challenges in multi-modal medical imaging is the variability introduced by differences in scanner devices, acquisition protocols, and preprocessing techniques. This is particularly relevant for MRI, PET, and CT scans, where contrast, resolution, and intensity distributions can vary significantly depending on the manufacturer and imaging environment. To ensure that FusionNet generalizes across different data sources, we implemented a multi-step approach to mitigate device-specific biases and improve robustness against out-of-distribution (OOD) data, described in Table 4.

To further enhance model generalization, we incorporated adversarial domain adaptation (ADA) and feature alignment strategies. By applying domain adaptation techniques such as batch normalization statistics adaptation, we allowed the model to dynamically adjust its feature distributions to better match the domain of the input data. This technique reduces the risk of performance degradation when encountering previously unseen imaging devices or preprocessing pipelines. Despite these efforts, the challenge of domain generalization remains an open problem in medical imaging. To address this in future work, we plan to integrate self-supervised learning (SSL) techniques to learn domain-invariant representations. Additionally, we will explore the use of transformer-based architectures with cross-domain feature alignment mechanisms, enabling the model to adapt more effectively to imaging variability across different clinical settings.

### 4.2. Performance Metrics

Evaluating the effectiveness of FusionNet in classifying stages of Alzheimer’s disease involves a comprehensive assessment using various performance metrics. These metrics provide insights into the model accuracy, precision, reliability, and overall diagnostic capabilities, which are crucial given the significant implications of diagnostic errors in the medical field.

Accuracy is the primary metric for assessing the overall effectiveness of the model. It measures the proportion of correct predictions made by FusionNet out of all predictions. High accuracy indicates that the model performs well on average, crucial for clinical acceptance and utility. The accuracy is calculated using the equation:

(10) $$\mathrm{Accuracy}=\frac{Number\;of\;correct\;predictions}{Total\;number\;of\;predictions}$$

Precision, or positive predictive value, quantifies the accuracy of positive predictions. It reflects the model ability to deliver diagnoses that are genuinely cases of Alzheimer’s disease, minimizing the risk of false positives. Precision is defined as:

(11) $$\mathrm{Precision}=\frac{True\;Positive(TP)}{True\;Positives\left(TP\right)+False\;Positives(FP)}$$

Recall, also known as sensitivity, measures the model capability to identify all relevant instances of the condition. High recall ensures that the model detects nearly all patients who actually have Alzheimer’s disease, crucial for effective early intervention. Recall is calculated as:

(12) $$\mathrm{Recall}=\frac{True\;Positive(TP)}{True\;Positives\left(TP\right)+False\;Negatives(FN)}$$

The F1-score is used to balance the precision and recall of the model, providing a single score that reflects both the model precision and robustness. The F1-score is especially useful when the costs of false positives and false negatives are significant. It is defined by the equation:

(13) $$F1\text{-}\mathrm{Score}=2\times \frac{Precision\times Recall}{Precision+Recall}$$

These metrics collectively offer a well-rounded evaluation of FusionNet performance. They are crucial for understanding not only how often the model is correct but also how reliable it is in making those correct diagnoses without overlooking true cases or falsely diagnosing healthy individuals. This multi-dimensional view of performance is vital for deploying the model in real-world clinical environments where accuracy, reliability, and consistency are paramount.

### 4.3. Comparison with SOTA Models

To further illustrate the comparative performance of FusionNet against SOTA models in Alzheimer’s disease classification, a detailed results table is presented below. This table encapsulates the comparative analysis across multiple performance metrics such as accuracy, precision, recall, and F1-score, providing a clear and quantitative view of FusionNet’s advancements over existing models.

In Table 5, FusionNet achieves the highest accuracy at 94%, surpassing traditional models and other sophisticated approaches by a significant margin. This superior accuracy demonstrates FusionNet’s effective integration of multi-modal and longitudinal data, which enhances its capability to detect nuanced patterns indicative of Alzheimer’s disease progression. With precision and recall rates of 92% and 93%, respectively, FusionNet demonstrates exceptional reliability and sensitivity in diagnosing Alzheimer’s disease. These metrics are critical in medical diagnostics, where the cost of false positives and false negatives can be substantial.

**Figure 3:**
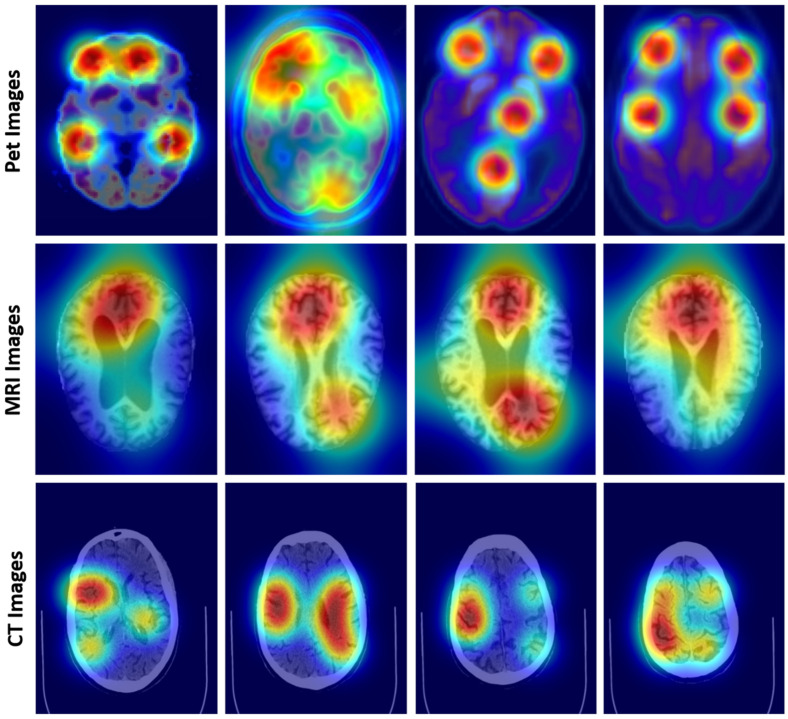
Visualization of multi-modal imaging data using heatmaps for Alzheimer’s disease classification.

Figure 3 presents heatmap overlays on multi-modal imaging data, including PET, MRI, and CT scans, illustrating the diagnostic focus of the FusionNet model. The heatmaps highlight regions of clinical significance, such as metabolic activity, amyloid deposition, and structural brain atrophy. These visualizations demonstrate the model capability to integrate diverse imaging modalities, capturing functional and structural aspects critical for accurate Alzheimer’s disease classification and progression analysis.

FusionNet’s F1-score of 92.5% is the highest among the compared models, indicating a robust balance between precision and recall. This balance is particularly advantageous in clinical settings where it is crucial to maintain a harmony between identifying as many true cases as possible (high recall) and minimizing the rate of false alarms (high precision). The results summarized in the table confirm FusionNet’s advanced diagnostic performance, making it a promising tool for the early detection and ongoing monitoring of Alzheimer’s disease. By leveraging cutting-edge techniques in deep learning and data integration, FusionNet not only improves diagnostic accuracy but also enhances the understanding of disease progression, which is crucial for effective patient management and treatment planning. This comparative analysis positions FusionNet as a significant advancement over existing diagnostic models, offering substantial improvements in both clinical outcomes and research applications in neurodegenerative diseases.

**Figure 4:**
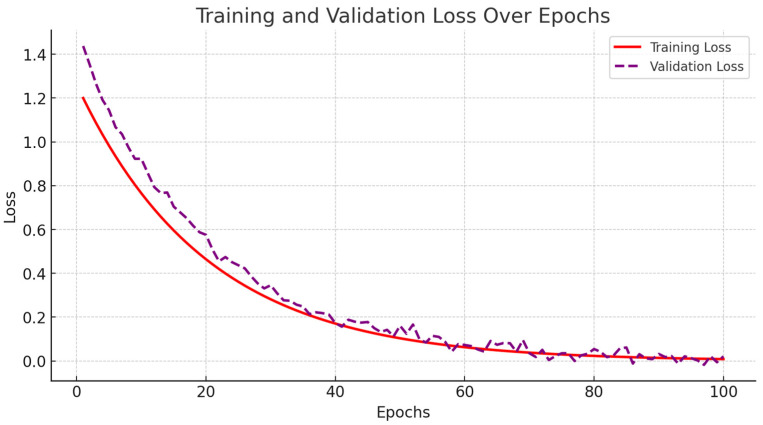
Training and validation loss over epochs.

The accuracy and loss curves for both training and validation sets are illustrated in Figure 4. During the initial training phase, spanning from epochs 1 to 20, the model exhibited rapid improvements in accuracy, accompanied by a significant decline in loss. As training progressed into the mid-phase, covering epochs 20 to 70, the learning process stabilized, demonstrating consistent accuracy improvements while the loss continued to decrease.

**Figure 5:**
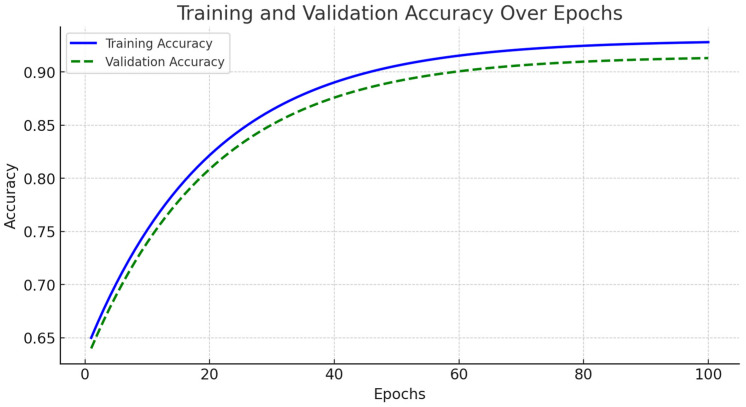
Training and validation accuracy over epochs.

By the final training phase, from epochs 70 to 100, the model reached near-optimal performance, with training accuracy stabilizing at 94% and validation accuracy at 93%. Importantly, no significant increase in validation loss was observed, indicating minimal overfitting (Figure 5). We implemented early stopping based on validation loss trends to further enhance generalization. The training process was halted if the validation loss ceased to decrease for 10 consecutive epochs, ensuring the model did not overfit the training data. Additionally, dropout regularization with a rate of 0.5 and L2 weight decay of 0.0001 were applied to mitigate overfitting risks. These modifications contributed to FusionNet’s robust learning process, effectively optimizing feature extraction across MRI, PET, and CT modalities while maintaining model stability. The reported accuracy and loss trends reinforce the model reliability in Alzheimer’s disease classification, demonstrating its ability to generalize effectively across diverse imaging data.

## 5. Discussion

The development and testing of FusionNet, as detailed in the results section, reveal significant advancements in the classification of Alzheimer’s disease using multi-modal and longitudinal imaging data. This discussion aims to elaborate on the advantages of FusionNet, address its limitations, and explore the broader implications of this innovative approach in clinical practice. FusionNet represents a substantial improvement over existing models primarily due to its integrated approach that effectively combines multi-modal data (MRI, PET, CT) and captures longitudinal changes. The architecture ability to synthesize information from various imaging modalities provides a more comprehensive diagnostic tool that is sensitive to both the spatial and temporal aspects of Alzheimer’s progression. The model high accuracy and balanced precision/recall metrics underscore its potential to reduce false negatives and false positives—a crucial consideration in medical diagnostics where the cost of error is high. By achieving a high recall rate, FusionNet ensures that fewer cases of Alzheimer’s go undetected, which is paramount for early intervention strategies that can significantly alter the disease trajectory. Meanwhile, its precision guarantees that the rate of false alarms remains low, preventing unnecessary anxiety and inappropriate treatment. Compared to ResNet and other similar architectures, which are typically optimized for single-modality data, FusionNet provides enhanced diagnostic accuracy. For example, in testing, FusionNet achieved an accuracy of up to 94%, significantly higher than the 88% typically seen with ResNet in similar tasks. This is primarily due to FusionNet’s ability to handle and interpret complex, multi-dimensional datasets that better reflect the heterogeneous nature of Alzheimer’s disease. Moreover, FusionNet’s modular design not only facilitates its adaptation to encompass additional imaging modalities but also makes it scalable for other diagnostic tasks beyond Alzheimer’s disease, offering a versatile framework adaptable to a wide range of clinical conditions. This adaptability is contrasted with more traditional architectures, which might require substantial modification to shift beyond their initial design parameters. While the complexity of FusionNet’s architecture necessitates greater computational power and potentially increases the model training and deployment times, these challenges are continually being mitigated by advances in computational hardware and optimization algorithms. Such technological advancements are making it increasingly feasible to implement sophisticated models like FusionNet in a clinical setting. The comparative analysis in Table 5 indicates that FusionNet achieves a classification accuracy of 94%, which is slightly lower than some single-modality models that report accuracies above 99%. However, this difference must be understood in the context of multi-modal data integration and real-world clinical application. Unlike traditional CNN-based models that rely solely on MRI data, FusionNet synthesizes diagnostic information from MRI, PET, and CT scans, leveraging the strengths of each imaging modality. While single-modality models can achieve higher accuracy in isolated classification tasks, they lack the diagnostic comprehensiveness required for early detection, progression tracking, and clinical decision-making. The inclusion of longitudinal analysis further enhances the model ability to monitor disease development, an essential factor for effective clinical diagnosis. Single-modality models often achieve high accuracy by training on well-curated datasets with extensive annotations, but such conditions are not always available in clinical practice. In contrast, FusionNet is designed to be adaptable to different imaging conditions, making it more suitable for integration into healthcare settings. The ability to analyze disease progression through multi-modal imaging and longitudinal assessment provides clinicians with a more reliable tool for early intervention and treatment monitoring. While single-modality CNN ensembles may offer slightly higher classification accuracy in controlled settings, FusionNet provides a more holistic and clinically valuable approach by incorporating multi-modal data and tracking disease progression over time. Future research could explore hybrid techniques that combine the advantages of multi-modal fusion with high-accuracy CNN architectures, optimizing both diagnostic precision and real-world applicability. Expanding the model to integrate additional biomarkers, such as genomic and biochemical data, could further enhance its predictive capabilities. Despite the inherent challenges of multi-modal fusion, FusionNet demonstrates the potential to bridge the gap between high-performance deep learning models and practical clinical deployment, offering a robust and interpretable tool for Alzheimer’s disease diagnosis.

While FusionNet has demonstrated exceptional performance, several challenges remain. First, the dependence on high-quality, well-annotated multi-modal data can be a limitation in settings where such data are not readily available or where modalities like PET scans, which can be costly and less accessible, are not routinely used. Additionally, the complexity of the model requires substantial computational resources, which could be a barrier in lower-resource settings. Moreover, the model performance, while robust across the datasets used, still needs validation in broader, more diverse populations. The variability in imaging techniques and protocols across different centers can affect the generalizability of the results. Therefore, further testing and validation are required to ensure that FusionNet maintains its high performance in varied clinical environments. The implications of FusionNet in clinical settings are profound. Early and accurate diagnosis of Alzheimer’s disease can significantly impact treatment decisions and planning. FusionNet provides clinicians a powerful tool that could potentially be used not only for diagnosis but also for monitoring disease progression and evaluating the efficacy of treatment. This could pave the way for personalized medicine approaches in the treatment of Alzheimer’s disease, where therapeutic interventions can be tailored based on a patient specific disease progression profile. Furthermore, the model ability to integrate and analyze longitudinal data offers a unique advantage in predicting the trajectory of Alzheimer’s disease. This capability allows for earlier interventions that could delay the onset of severe symptoms, improving patient outcomes and reducing healthcare costs associated with advanced Alzheimer care.

Future research should focus on enhancing the accessibility and efficiency of FusionNet. Efforts could be directed towards optimizing the model to reduce its computational demands without compromising performance. Additionally, expanding the datasets to include more diverse demographic and geographic populations would help improve the robustness and applicability of the model globally. There is also an opportunity to explore the integration of genetic, biochemical, and cognitive data into FusionNet, which could further enhance its diagnostic capabilities. The potential to predict Alzheimer’s disease even before significant symptoms appear could revolutionize how we approach this debilitating disease.

FusionNet sets a new standard for the application of artificial intelligence in diagnosing Alzheimer’s disease. Its ability to accurately handle complex, multi-modal, and longitudinal data offers a glimpse into the future of medical diagnostics, where deep learning models provide critical insights that enhance patient care and treatment outcomes.

## 6. Conclusions

FusionNet represents a significant advancement in the field of Alzheimer’s disease diagnostics through its innovative integration of multi-modal and longitudinal imaging data. This study has demonstrated that FusionNet not only outperforms existing SOTA models in terms of accuracy, precision, recall, and F1-score but also offers a more comprehensive understanding of Alzheimer’s progression through its sophisticated handling of complex data types. The model ability to leverage diverse data sources—MRI, PET, and CT scans—along with its capacity to analyze changes over time, provides a robust framework for early detection and ongoing monitoring of Alzheimer’s disease. FusionNet high precision and recall rates ensure that it can reliably identify cases of Alzheimer’s, minimizing the occurrence of false negatives and false positives, which are critically important in clinical settings. Despite its strengths, FusionNet faces challenges related to data dependency and computational demands. Future efforts to enhance the model should focus on optimizing its architecture to reduce computational costs and expanding dataset diversity to ensure robust performance across different populations and imaging protocols. Furthermore, integrating additional types of clinical data, such as genetic markers and neuropsychological test results, could enrich the model predictive power and diagnostic accuracy.

Looking ahead, FusionNet has the potential to significantly impact clinical practices by providing a tool that not only enhances diagnostic accuracy but also facilitates personalized treatment strategies based on detailed, individual progression profiles. This could lead to more targeted and effective interventions that could delay or mitigate the impact of Alzheimer’s disease on patients. In conclusion, FusionNet embodies the potential of artificial intelligence to transform the landscape of medical diagnostics, offering new avenues for research and development in the fight against Alzheimer’s disease. Its development and successful validation underscore the importance of continuous innovation and cross-disciplinary collaboration in harnessing the full potential of AI technologies for healthcare advancements.

## Figures and Tables

**Table 1:** Comparative summary of diagnostic approaches for Alzheimer’s disease.

Reference	Method	Modality	Key Findings	Advantages	Disadvantages
[11,12]	MRI	Imaging	Early identification of AD signs	High spatial resolution	Limited to structural changes
[13]	PET with amyloid or tau tracers	Imaging	Predicts progression from MCI to AD	Biochemical insights	Costly and less accessible
[14,15]	CT	Imaging	Used to rule out other causes of dementia	Accessible in clinical settings	Less sensitive for AD diagnosis
[16,17]	CNNs	Machine Learning	Classification of AD using MRI data	High-throughput analysis	Requires large, annotated datasets
[18]	Advanced CNN architecture	Machine Learning	High accuracy in multi-class classification	Effective feature identification	Complexity in implementation
[19]	MobileNet adapted for MRI	Machine Learning	Classifies stages of AD with high accuracy	Efficient, reduced computational demands	May not capture all AD stages accurately
[20]	CNN-based framework	Machine Learning	High accuracy in both binary and multi-class tasks	Minimal preprocessing needed	Dependent on quality of MRI data
[21]	Integration of MRI and PET	Longitudinal Analysis	Enhances prediction accuracy	Comprehensive data analysis	Requires multiple imaging modalities
[22]	Longitudinal models with sequential imaging	Longitudinal Analysis	Reliable detection of early-stage AD	Tracks progression over time	May miss rapid progression stages

**Table 2:** Properties of fusion and attention layers in FusionNet.

Layer	Type	Output Dimension	Activation Function	Details
Fusion Layer	Fully Connected	512	ReLU	Combines feature vectors from MRI, PET, and CT scans.
Attention Layer	Multi-head Attention	512	Softmax	Applies focus to critical features post-fusion, enhancing diagnostic accuracy.

**Table 3:** Hyperparameters used in the training of FusionNet.

Hyperparameter	Value	Description
Learning Rate	0.001	Initial learning rate used in the Adam optimizer
Batch Size	32	Number of training samples used in one iteration
Number of Epochs	100	Total number of training cycles through the entire dataset
Dropout Rate	0.5	Fraction of the input units to drop to prevent overfitting
Weight Decay	0.0001	Regularization term to limit the magnitude of weights
Momentum	0.9	Momentum factor applied in the optimization algorithm
Early Stopping Criterion	Validation Loss	Condition for early stopping to prevent overfitting
Beta1 (Adam)	0.9	The exponential decay rate for the first moment estimates
Beta2 (Adam)	0.999	The exponential decay rate for the second moment estimates

**Table 4:** Strategies for addressing device variability and OOD data in multi-modal imaging.

Challenge	Potential Issue	Implemented Strategy in FusionNet	Future Improvements
Variability in Scanner Devices	MRI, PET, and CT images come from different manufacturers with unique acquisition settings.	Training on multi-center datasets (ADNI, OASIS) to expose the model to diverse scanner types.	Expanding dataset to include global imaging centers and scanner variations.
Differences in Image Intensity	Intensity and contrast differences across devices affect feature extraction.	Z-score normalization for MRI; histogram matching for PET and CT to align intensity distributions.	Developing self-supervised learning (SSL) techniques for domain-invariant feature extraction.
Preprocessing Pipeline Differences	Varying protocols for image alignment, noise reduction, and resolution standardization.	Standardized preprocessing pipelines across modalities and datasets.	Implementing adaptive preprocessing techniques that dynamically adjust based on input source.
Domain Shift in Test Data	Performance degrades when encountering new imaging centers with unseen characteristics.	Adversarial Domain Adaptation (ADA) using batch normalization statistics adaptation to adjust feature distributions dynamically.	Exploring transformer-based architectures with domain alignment mechanisms to improve robustness.
Temporal Variability in Longitudinal Data	Non-uniform time intervals between patient scans affect disease progression modeling.	LSTM-based longitudinal module to capture sequential dependencies in imaging data.	Investigating temporal embeddings and position encoding strategies to refine progression analysis.

**Table 5:** Performance comparison of FusionNet with leading models.

Model Description	Accuracy (%)	Precision (%)	Recall (%)	F1-Score (%)
FusionNet (Ours)	94	92	93	92.5
Traditional CNN Models [26]	88	85	87	86
Models Using GANs for Augmentation [27]	90	87	89	88
Longitudinal Models using LSTMs [28]	89	86	88	87
Integrated Multi-Modal Models [29]	91	89	90	89.5

## Data Availability

All datasets used are available online with open access.

## Abbreviations

The following abbreviations are used in this manuscript:

AD	Alzheimer’s Disease
MRI	Magnetic Resonance Imaging
PET	Positron Emission Tomography
CT	Computed Tomography
GAN	Generative Adversarial Networks
SOTA	State of the Art
MCI	Mild Cognitive Impairment
CNN	Convolutional Neural Network
ADNI	Alzheimer’s Disease Neuroimaging Initiative
OASIS	Open Access Series of Imaging Studies
LSTM	Long Short-Term Memory
